# Bone evaluation study-2: update on the epidemiology of osteoporosis in Germany

**DOI:** 10.1007/s11657-024-01380-9

**Published:** 2024-04-09

**Authors:** Peyman Hadji, Elizabeth Esterberg, Dominik Obermüller, Robert Bartsch

**Affiliations:** 1 Frankfurter Hormon- Und Osteoporosezentrum, Frankfurt Am Main, Germany; 2 Philipps-Universität Marburg, Marburg, Germany; 3 RTI Health Solutions, Research Triangle Park, NC USA; 4 Institute for Applied Health Research Berlin GmbH, Berlin, Germany; 5 Amgen GmbH, Munich, Germany

**Keywords:** Osteoporosis, Prevalence, Fracture, Care gap, Secondary analysis

## Abstract

**Summary:**

Osteoporosis is the most common bone disorder. Our data gives an estimate of around 5.87 million cases of osteoporosis in the general German population in 2018. Only 30% of insured individuals who suffered an osteoporotic fracture and/or had a confirmed diagnosis of osteoporosis, received an appropriate prescription.

**Purpose:**

Osteoporosis is the most common bone disorder. It particularly affects elderly people and increases the risk of atraumatic fractures. The aim of this study was to estimate the prevalence of osteoporosis in the general German population aged ≥ 50 years and to collect data on the frequency of prescription of osteoporosis-specific medication in order to assess the treatment gap.

**Methods:**

Retrospective analysis of anonymized data of individuals aged ≥ 50 years insured under statutory healthcare schemes from the database of the Institute for Applied Health Research Berlin (InGef) for 2018 (study population). Insured individuals with osteoporosis were identified based on osteoporosis diagnoses, osteoporosis-specific prescriptions, or osteoporotic fractures. Thus, we estimated the prevalence of osteoporosis in the general German population aged ≥ 50 years. The prevalence of diagnoses, fractures, and prescriptions was determined for the study population and stratified by age and gender.

**Results:**

Within the study population of 1,599,299 insured individuals, a prevalence of osteoporosis of 15.9% was determined. This estimated approximately 5.87 million cases of osteoporosis for the general German population. 81.6% of the cases were women. Osteoporosis-specific prescriptions were received by 30.0% of the insured individuals in the study population who had been diagnosed with osteoporosis and/or suffered an osteoporotic fracture.

**Conclusions:**

Germany has a high prevalence of osteoporosis. Only a small portion of individuals who may require osteoporosis-specific treatment actually receive it.

**Supplementary Information:**

The online version contains supplementary material available at 10.1007/s11657-024-01380-9.

## Introduction

Osteoporosis is the most common bone disease in humans. It leads to reduced bone mass and density, as well as a deterioration of the microarchitecture leading to an increased risk of atraumatic fractures [1].

Various studies have examined the prevalence of osteoporosis in Germany. The Bone Evaluation Study (BEST) found a prevalence of 14% for all individuals over 50 years of age in 2009. In this study, the prevalence was 24% for women and 6% for men [2]. The Scorecard for Osteoporosis in Europe (SCOPE) determined the prevalence of osteoporosis in people aged 50 and over in the countries of the European Union for the year 2019. According to this study, the prevalence in Germany was 22.6% for women and 6.6% for men of the same age. This placed Germany second within Europe [3]. An examination by the Robert Koch Institute revealed an osteoporosis prevalence of 24.0% in women aged 65 and over, and 5.6% in men of the same age group [4]. Moreover, the disease burden due to osteoporotic fractures, as measured by the parameter “disability-adjusted life years”, is high in Germany. According to an international study published in 2022, Germany is among the top five countries with the highest prevalence of death due to osteoporosis-related fractures [5].

In addition to the high prevalence, there is a significant treatment gap provided to patients with osteoporosis in Germany. The large number of multiple fractures documented in the BEST study suggests that not all diagnosis and treatment options have been fully utilized to prevent fractures [2]. Further studies support this outcome: the SCOPE study reported a treatment gap of 76% for individuals aged ≥ 50 [3], and another study published in 2021 reported a treatment gap of 91% for women aged ≥ 70 in Germany [6].

In Germany, the treatment of osteoporosis patients is primarily based on the guidelines of the governing body of German osteology (Dachverband Osteologie e. V). These guidelines recommend specific medication therapy, among other interventions, for all patients who have already suffered an osteoporotic fracture of vertebral bodies or the neck of the femur, as they are at very high risk of subsequent fractures. In addition to medical treatment, general measures for the prevention of fractures should also be implemented for all individuals at risk [7].

The increasing number of osteoporotic fractures also has consequences for public health expenditures. In Germany alone, osteoporosis results in annual costs amounting to 13.8 billion euros [3]. This justifies the development of a Disease Management Program for Osteoporosis, which has been commissioned by the Federal Joint Committee and is currently in the process of implementation [8].

In 2013, the BEST study revealed a high prevalence of osteoporosis and a large treatment gap in the care of osteoporosis patients in Germany. The aim of the Bone Evaluation Study-2 (BEST-2) was to assess the status in Germany nearly 10 years after the publication of the original BEST data in order to be able to evaluate changes, particularly with regard to the prevalence of osteoporosis, as well as to the epidemiological characteristics and the healthcare situation in the German population aged ≥ 50 years. In particular, we analyzed the entirety of prevalence of osteoporotic fractures, osteoporosis diagnoses, and osteoporosis-specific prescriptions of patients ≥ 50 years in Germany.

## Methods

### Study design

**Fig. 1:**
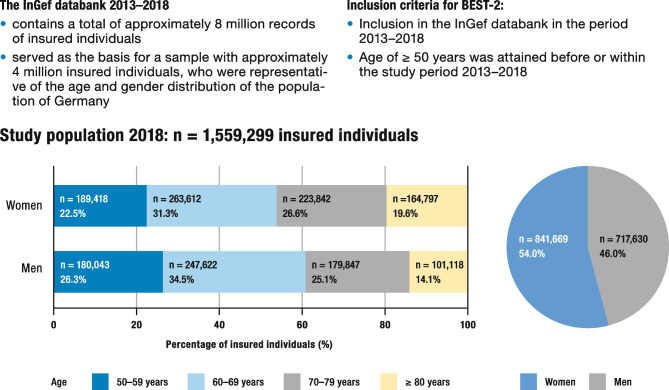
Insured individuals from the InGef database who were included in this analysis. Gender share and age distribution in the study population for the year 2018. Adapted from [1]

The BEST-2 study was performed as a cohort study based on the InGef database [9]. This anonymized research database from the Institute of Applied Health Research Berlin contains longitudinal data from approximately 8.8 million individuals insured with German statutory health insurance companies. From this database, a sample of approximately 4 million insured individuals was drawn, who were representative with regard to age and gender for the total German population [9]. In this sample, insured individuals who met the inclusion criteria for the BEST-2 study for the years 2013–2018 were identified (Fig. 1).

### Participants

The study included the data of all insured individuals who were enrolled in the InGef database in the period from January 1, 2013 to December 31, 2018, and who were at least 50 years old at any point during the study period (Fig. 1). Patients with evidence of the following conditions during the baseline period were excluded from the analysis: "Osteogenesis imperfecta", "Paget disease", "Primary or secondary, benign or malignant bone tumor (C40*, C41*, C79.5).

### Outcomes

The BEST-2 study aimed to provide insight into various key characteristics related to osteoporosis.

In order to estimate the prevalence of osteoporosis for the entire German population aged 50 years and older, the study initially identified all insured individuals in the study population who had at least one of the following 3 indications of osteoporosis: the prescription of an osteoporosis-specific medication, an osteoporotic fracture, or a diagnosed case of osteoporosis. The prevalence of insured individuals with osteoporosis in the study population, thus determined, was then used to determine the prevalence of osteoporosis in the overall German population aged ≥ 50 years in the year 2018.

Within the study population, the prevalence of the diagnosis of osteoporosis was determined using the coded diagnosis codes of the International Classification of Diseases, 10th Revision, German Modification (ICD-10-GM).

In order to ascertain the prevalence of osteoporotic fractures within the study population, all fractures that occurred from the age of 50 onwards were initially considered as osteoporosis-related. These fractures were further narrowed down using ICD-10-GM codes, which were assigned by the respective treating physician. The included codes were additionally reviewed by osteoporosis experts who provided their approval for each fracture type in this study.

Among those study participants who suffered an osteoporotic fracture, the prevalence of the diagnosis of osteoporosis was also determined.

The prevalence of osteoporosis-specific prescriptions within the study population for the year 2018, among those who received an osteoporosis diagnosis and/or suffered an osteoporotic fracture, was identified based on the codes from the Anatomical Therapeutic Chemical (ATC) Classification System and confirmed by experts.

The included ICD-10 and ATC codes are summarized in Tables S1 and S2, respectively (Internet Supplement).

### Stratified analysis

The prevalences of osteoporotic fractures, diagnosis of osteoporosis, and osteoporosis-specific prescriptions were stratified in age and gender groups, as illustrated in Fig. 1. Participants were included in the calculation of the age group that corresponded to their age on January 1, 2018. The age groups were defined as follows: 50–59 years, 60–69 years, 70–79 years, and ≥ 80 years.

### Statistics

The evaluation of the results was performed using descriptive statistical methods. All analyses were conducted by InGef staff using R, Version 4.0.2. All study measures were analyzed descriptively through tabular and graphical displays of mean values, standard deviations medians, and ranges of continuous variables of interest and frequency distributions for categorical variables. Prevalence of osteoporosis in 2018 was calculated as the number of individuals in 2018 who have evidence of osteoporosis and who are ≥ 50 years of age during 2018, divided by the annual population at risk (i. e., the total number of adults ≥ 50 years of age at any time during the study year, as reflected in the InGef database).

### Ethics

All data from insured individuals and healthcare providers in the InGef database were anonymized in accordance with German data protection regulations. Since the use of databases for health services research is in compliance with German legislation, no further approval was required for the conduct of this study. Because only anonymized data were used, no declaration of consent from the insured individuals was necessary.

## Results

### Study population

In total, the data from 1,555,299 insured individuals from the InGef database were included in the study population in the year 2018. Of these, 54% were women. The average age of women was 69.4 years (± 10.5 years), and men had an average age of 67.6 years (± 9.8 years). The age group of 60 to 69-year-olds represented the largest proportion of the study population, accounting for 31.3% of women and 34.5% of men. A detailed overview of the study population for the year 2018 is represented in Fig. 1. The study participants had the following prevalence of risk factors for osteoporosis: 9.6% of the participants were taking glucocorticoids, 2.4% were underweight, 0.6% of the participating women were taking aromatase inhibitors, and 0.5% had major osteoporotic fractures.

### Prevalence of osteoporosis diagnoses, osteoporotic fractures and osteoporosis-specific prescriptions in the study population in the year 2018

**Fig. 2:**
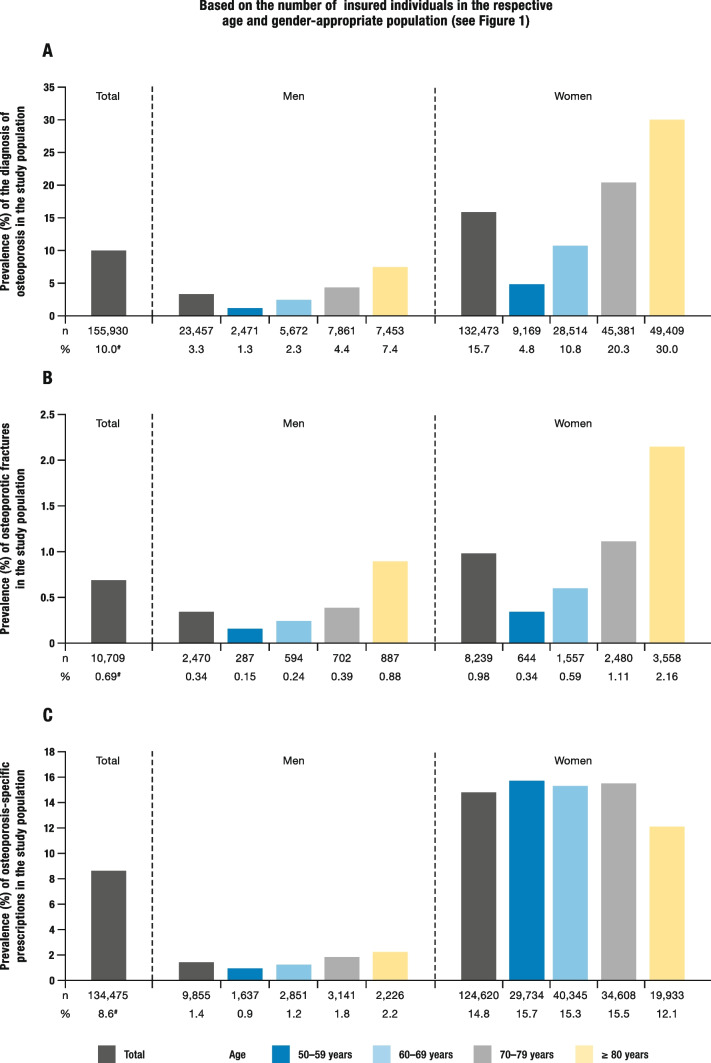
Key characteristics of the study population for the year 2018, stratified by age and gender. **A** Prevalence of osteoporosis diagnosis. **B** Prevalence of osteoporotic fractures. **C** Prevalence of osteoporosis-specific prescriptions. **A**–**C** The stratified data are relative to the number of insured individuals in the age and gender-appropriate population (see Fig. 1). # Based on n = 1,559,299. **A** and **B** modified from [1]

In 2018, osteoporosis was diagnosed in a total of 10% of insured individuals (155,930). Among them, 132,473 diagnoses (85.0%) were women, and 23,457 diagnoses (15.0%) were men. The frequency of these diagnoses increased with age (Fig. 2A).

A total of 10,709 insured individuals (0.7%) suffered an osteoporotic fracture. The largest proportion of these fractures (33.2%) occurred in women over 80 years of age. The prevalence of osteoporotic fractures increased with age for both men and women (Fig. 2B).

In total 134,475 insured individuals (8.6%) received an osteoporosis-specific prescription in 2018. Of these prescriptions, 9,855 (7.3%) were issued for men. For women, it was almost 13 times higher: 124,620 (92.7%; Fig. 2C).

### Estimated prevalence of osteoporosis in the German population aged 50 and over

**Fig. 3:**
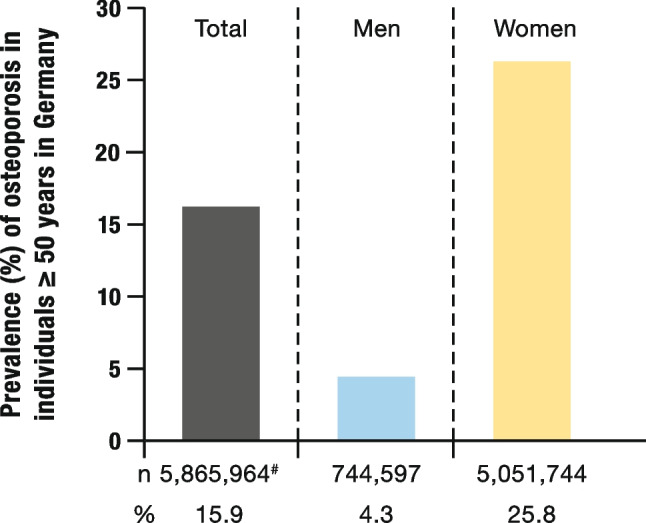
Prevalence of osteoporosis, estimated for the German population ≥ 50 years for the year 2018. The following cases were considered as osteoporosis: individuals with osteoporosis = osteoporosis-specific prescription and/or the diagnosis of an osteoporotic fracture and/or the diagnosis of osteoporosis. The prevalence in the total population was determined based on the number of individuals aged ≥ 50 years, who were living in Germany in 2018. Total: n = 36,851,612; men: n = 17,283,769; women: n = 19,567,843. ^#^ The case numbers of men and women add up to 5,796,341 cases. This deviation from the total number arises from the calculation method used and the rounding effects. Modified from [1]

The presented prevalences indicate that in 2018, 248,206 insured individuals (15.9%) of the study population (diagnosis, fracture or prescription) had osteoporosis. This included 217,290 women and 30,916 men. In the age groups, the highest prevalence, regardless of gender, was found in the 70–79 age group (50–59: 16.19%; 60–69: 27.2%; 70–79: 30.03%; > 80: 26.58%). Based on the recorded prevalence, the total number of individuals aged ≥ 50 years in Germany was estimated. Consequently, there was a total of 5,865,964 cases of osteoporosis. Among those affected, 5,051,744 were women and 744,597 were men (Fig. 3).

### Prevalence of the diagnosis of osteoporosis among individuals who had suffered an osteoporotic fracture

**Fig. 4:**
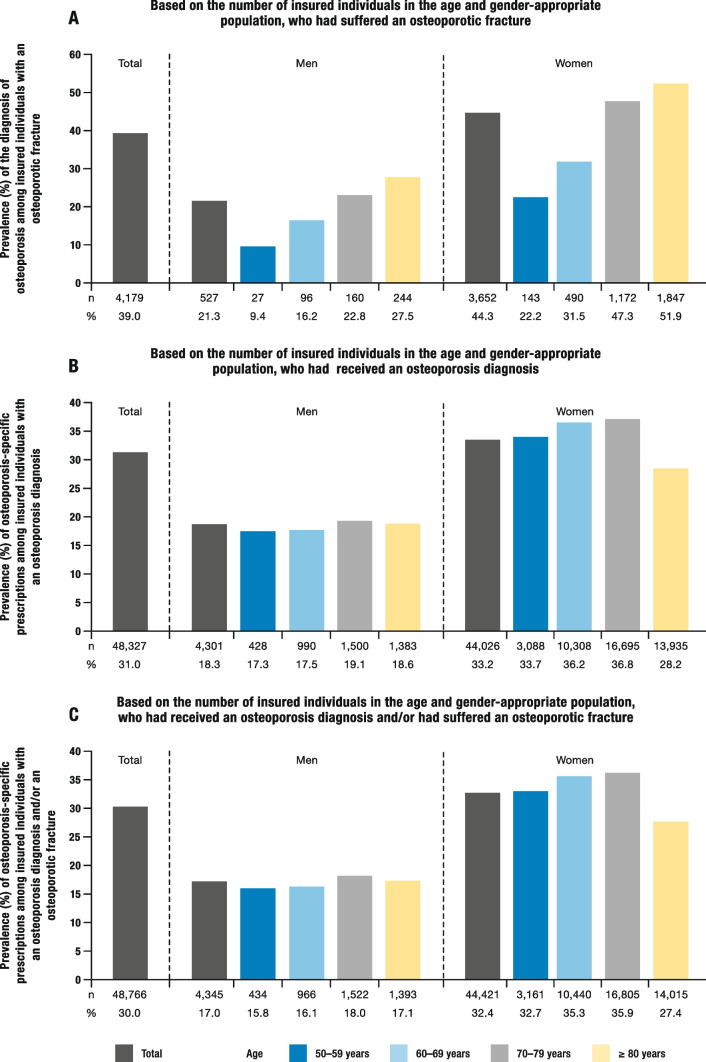
Additional key characteristics of the study population for the year 2018, stratified by age and gender. **A** Prevalence of osteoporosis diagnoses among insured individuals who suffered an osteoporosis-specific fracture. **B** Prevalence of osteoporosis-specific prescriptions among insured individuals with an osteoporosis diagnosis. **C** Prevalence of osteoporosis-specific prescriptions among insured individuals with an osteoporosis diagnosis and/or an osteoporotic fracture. **A** and **C** adapted from [1]

Among a total of 10,709 individuals in the study population, who had suffered an osteoporosis-related fracture in 2018, the diagnosis of osteoporosis was only made in 39.0% (4,179 cases). Women received this diagnosis nearly 7 times more often than men (3,652 cases vs. 527 cases). The frequency of an osteoporosis diagnosis among individuals with an osteoporotic fracture progressed with increasing age, regardless of gender (Fig. 4A).

### Prevalence of osteoporosis-specific prescriptions among those with an osteoporosis diagnosis and/or an osteoporotic fracture

In the study population, just around one-third (31.0%; 48,327 out of 155,930) of the insured individuals, who had been diagnosed with osteoporosis in 2018, received an osteoporosis-specific prescription. After an osteoporosis diagnosis, women received such prescriptions in 33.2% of cases (44,026 out of 132,473), while men received them in 18.3% of cases (4,301 out of 23,457; Fig. 4B).

Among those participants who had suffered an osteoporosis-related fracture in 2018, 19.5% (2,323 out of 10,709) received an osteoporosis-specific prescription. Women received such prescriptions in 25.3% of cases (2,084 out of 8,239), while men received them in 9.7% of cases (239 out of 2,470).

When combining the osteoporosis-specific prescriptions for those who had received an osteoporosis diagnosis with those who had suffered an osteoporosis-related fracture, a total of 30.0% of study participants (48,766 out of 162,740) received osteoporosis-specific prescriptions in 2018. Women received these prescriptions in 32.4% of cases (44,421 out of 137,227), while men received them in 17.0% of cases (4,345 out of 25,513). Osteoporosis-specific prescriptions were fairly evenly distributed across all age groups for both men and women. Only the group of women aged over 80 received these prescriptions slightly less frequent than women in the other age groups (Fig. 4C).

## Discussion

The results of the BEST-2 study provide an updated assessment of the prevalence of osteoporosis, osteoporotic fractures and osteoporosis-specific prescriptions in the German population aged ≥ 50 for the year 2018. Estimated for the German population ≥ 50 years, there are 5.87 million people who are affected by osteoporosis in Germany. Of these, 744,597 were men and 5,051,744 were women.

In recent years, several studies have been published that examined the prevalence of osteoporosis in Germany. The BEST study calculated an osteoporosis prevalence of 14% for the study population aged ≥ 50 for the year 2009 [2]. In our recent analysis for the year 2018, we were able to observe a slight increase to 15.9%. This difference could, in part, be due to the different datasets underlying the two studies. While the BEST study was based on data from the Techniker Krankenkasse health insurance provider, the BEST-2 study used data from the InGef database. Furthermore, the BEST study applied correction based on Brecht and Schädlich, which classified fewer fractures as potentially osteoporosis-related. However, this correction may have also led to an underestimation of the actual prevalence. No correction based on Brecht and Schädlich was applied to the BEST-2 study, so a certain overestimation of the prevalence is possible here.

Another study estimated a prevalence of 3.49 million osteoporosis patients aged 50 and over in Germany for the year 2016 [10]; this value is significantly lower than the 5.87 million affected individuals identified in our study. However, this report only considered patients who had received an osteoporosis diagnosis. Osteoporotic fractures and osteoporosis-specific prescriptions were not taken into account [10].

The SCOPE 2021 study calculated a prevalence of 22.6% for women aged ≥ 50 in Germany for the year 2019. For men, the prevalence was reported to be 6.6%. The total number of individuals with osteoporosis was estimated at 5.66 million [3]. The slight variations with our results may have methodological reasons as the prevalence of osteoporosis in the SCOPE study was determined based on the bone density of the neck of the femur. A study published in 2017 also examined the prevalence of osteoporosis in Germany, based on self-reports. In this study 24.0% of women aged 65 and over reported suffering from osteoporosis, while for men it was 5.6% [11]. These results are also comparable to the findings of the BEST-2 study.

Our studies and those mentioned above show a gender-specific difference in the prevalence of osteoporosis. The reported prevalence of women and men is 4:1. The ratio of diagnoses in our study population is 7:1, suggesting under-reporting or limited access to healthcare for men. If so, the data for men may be underestimated.

Overall, the prevalence of osteoporosis diagnosis, osteoporotic fractures and osteoporosis-specific prescriptions is higher in women than in men in every age group. This can be attributed to several factors. One of these is hormonal differences. Estrogen plays a crucial role in maintaining bone density, and its decline during menopause in women is a well-established risk factor for osteoporosis [12]. Men, on the other hand, experience a more gradual decline in testosterone, which has a less pronounced effect on bone health [13]. Another factor are differences in bone mineral density. Women typically reach peak bone mass earlier in life than men. If the peak bone mass is lower, there's a higher likelihood of developing osteoporosis later in life [14]. This discrepancy could contribute to the higher prevalence in women. Furthermore, women may be more proactive in seeking healthcare, leading to higher rates of osteoporosis diagnosis. Men, on the other hand, might be less likely to seek medical attention for bone health issues until symptoms become more severe [15]. In addition, osteoporosis is often perceived as a women's health issue, leading to underdiagnosis in men. Healthcare providers may be less likely to consider osteoporosis in their male patients, resulting in fewer diagnoses and prescriptions [16].

With regard to osteoporosis-specific treatment, there still appear to be a significant treatment gap in Germany. Less than one-third of insured individuals (30.0%) who had suffered an osteoporosis-related fracture and/or had been diagnosed with osteoporosis, received appropriate treatment. This could be explained by the low number of osteoporosis diagnoses among individuals with an osteoporosis-related fracture. Among 10,709 patients who had suffered an osteoporosis-related fracture in 2018, only 39% received an osteoporosis diagnosis. However, there seem to have been improvements in recent years. In 2009, only 18.9% of individuals with an osteoporosis-related fracture were diagnosed with osteoporosis [2]. At that time, 28% of insured individuals with an osteoporosis diagnosis received treatment and 21% of individuals, who had suffered a first fracture, received treatment [2] – i.e. less than in the current study (30%).

The treatment gap was larger in men than in women. Whilst just around one third of women (33.2%) received an appropriate prescription following an osteoporosis diagnosis, this was only the case for less than one in five men (18.3%). After an osteoporotic fracture, less than 10% of men received a specific medication, compared to one in four women (25.3%). The fact that osteoporosis in men with fractures is often not diagnosed [17] could explain why the treatment gap is larger in men than in women.

Other studies also reported a treatment gap for osteoporosis in Germany. The SCOPE 2021 study found that in 2019 more than three-quarters (76%) of women, who needed osteoporosis treatment, did not receive it. Aside from declining numbers in the measurement of bone mineral density, the authors of the SCOPE study cited the fear of potential side-effects of bisphosphonate therapy as a possible cause [3]. For the year 2016 another study determined that only 36.9% of all osteoporosis patients in Germany, who had suffered an osteoporotic fracture, received osteoporosis treatment [10]. An analysis of data from insured individuals aged ≥ 70 years in the InGef database for the years 2011–2016 found that 85% of first fractures in these insured individuals remained untreated [18]. McCloskey and colleagues described a treatment gap of 91% for women aged 70 and older in Germany, despite most women having an increased risk of fractures. They attributed this fact to the low diagnosis rate [6]. In a retrospective study of older patients, who had bone density measured prior to total hip replacement, osteoporosis was diagnosed in 18% of these patients. This had been previously diagnosed in only 27% of cases, and as a result only 37% of those affected received vitamin D supplementation, and only 22% received specific therapy [19]. The results of another study also point to a significant gap in the diagnosis of osteoporotic fractures. Routinely performed computed tomography (CT) scans of all patients aged 45 and over were examined for osteoporosis-related vertebral fractures, with their medical records going back at least 5 years. Such fractures were found in approximately 30% of the included patients. However, they were mentioned in only 25% of cases in the CT report [20].

Our study also has limitations. The inclusion of insured individuals based on the ICD-10-GM codes can be subject to coding errors and incorrect diagnoses. This is a fundamental issue in all database analyses, which we addressed by applying strict selection criteria for the included ICD-10-GM codes for osteoporosis, osteoporotic fractures and osteoporosis-specific prescriptions. Further, social determinants of health were not available in this database but are not expected to bias the results given the universal health system available in Germany. In addition, for outpatient diagnoses, there had to be at least two diagnoses, while for inpatient diagnoses a primary or secondary diagnosis was sufficient. The evidence of osteoporosis based on the prescription of osteoporosis-specific medications could have been influenced by the prescription of these medications for patients with an oncological disease. In order to minimize this error, insured individuals who received oncological therapy based on the ICD-10-GM codes C40, C41 and C79.5 were excluded from our analysis. Furthermore, medications that are additionally used for oncological indications are prescribed in different dosages, which also allowed for the exclusion of oncological patients. Since all analyses are based on the coding of the respective osteoporosis diagnosis, fracture, or treatment, the number of patients with diagnosed osteoporosis may have been underestimated. The InGef database only captures data from statutory health insurance providers. Screenings and treatments outside of this system were not recorded. Furthermore, in some cases, the fractures considered potentially osteoporosis-related may have had other causes. As already mentioned, no correction based on Brecht and Schädlich was carried out as part of BEST-2, so that an overestimation of prevalence is possible. Due to the different healthcare context of the countries USA and Germany, there are possible distortions when transferring the US distribution to the German BEST-2 patient population. Furthermore, the different healthcare context between the years 1986 and 2018 leads to possible distortions when transferring the distribution from 1986 to the BEST-2 patient population of 2018. In addition, the original study by Phillips et al. 1988 only gives the result of the survey of the 5 US experts [21]. A methodical description of how the distinction between fractures was made is not shown. It is therefore not clear how the sometimes very small differences between the fractures in the distribution came about. Brecht and Schädlich used a different diagnostic coding scheme (ICD-9 by Brecht and Schädlich [21]) compared to ICD-10-GM 2018 (BEST-2 study), which often cannot be translated directly. Finally, the distribution of men based on Brecht and Schädlich was not obtained from the expert survey of the original study by Phillips et al. 1988, but only from an assumption made in the study by Pientka et al. 1996: “Since the expert estimates only refer to women, we have set the proportions for men 10 percent lower than for women.” [22]. Overall, the assumption made in the BEST-2 study that 100% of the respective fractures correspond to osteoporosis will lead to an overestimation. The selection of fractures used in the study was therefore made prior to the study with experts in osteoporosis care with regard to the greatest possible association with osteoporosis. The extent of overestimation and thus bias is therefore considered to be low.

Nevertheless, the results of the BEST-2 study allow insights into the epidemiology of osteoporosis in Germany since the data used from the InGef database are representative of Germany with regard to age and gender and can therefore be extrapolated to the broader German population [9]. The results of the BEST-2 study reinforce the need for the immediate practical implementation of the Disease Management Program for Osteoporosis approved by the Federal Joint Committee and the Federal Ministry of Health [8].

## Conclusion

The results of the BEST-2 study demonstrate that in Germany, nearly 10 years after the publication of the BEST study, there is still a high prevalence of osteoporosis and a significant gap in the diagnosis and treatment of osteoporosis.

## Supplementary Information

Below is the link to the electronic supplementary material.

Supplementary file1 (PDF 179 KB)

## Data Availability

The data utilized in our study are not openly available due to legal and ethical constraints (sick funds data).
