# Correction: Bone evaluation study-2: update on the epidemiology of osteoporosis in Germany

**DOI:** 10.1007/s11657-024-01388-1

**Published:** 2024-04-23

**Authors:** Peyman Hadji, Elizabeth Esterberg, Dominik Obermüller, Robert Bartsch

**Affiliations:** 1 Frankfurter Hormon-Und Osteoporosezentrum, Frankfurt Am Main, Germany; 2 Philipps-Universität Marburg, Marburg, Germany; 3 RTI Health Solutions, Research Triangle Park, NC USA; 4 Institute for Applied Health Research Berlin GmbH, Berlin, Germany; 5 Amgen GmbH, Munich, Germany

**Correction: Archives of Osteoporosis (2024) 19:26**

10.1007/s11657-024-01380-9

In the original version of this article, the given and family names of Peyman Hadji, Elizabeth Esterberg, Dominik Obermüller, and Robert Bartsch were incorrectly structured. The name was displayed correctly in all versions at the time of publication.

The original article has been corrected.

