# Supplementary material for: Bone evaluation study-2: update on the epidemiology of osteoporosis in Germany
Source: Arch Osteoporos. 2024 Apr 9;19(1):26. doi: 10.1007/s11657-024-01380-9 (PMC11003882; doi:10.1007/s11657-024-01380-9)
Supplement: Supplementary file 1 — Supplementary file1 (PDF 179 KB) [file 11657_2024_1380_MOESM1_ESM.pdf]

Table S1: Diagnosis codes used for identification from the International Classification of Diseases, 10th Edition, German Modification (ICD-10-GM)

Number	Description	ICD-10-GM Code
	**Osteoporosis**	
1	Osteoporosis with current pathological fracture	M80.x, M48.5*
2	Osteoporosis without current pathological fracture	M81.x
	**Fractures**	
3	Vertebral fracture	S22.0x, S22.1<br>S32.0x<br>M80.x8*, M48.5*
4	Hip fracture	S72.0x except S72.08<br>S72.1, S72.2<br>M 80.x5*
5	Non-hip fracture, non-vertebral fractures	(S.22.3 (ribs))<br>S32.1, S32.4, S32.5 (pelvis)<br>S42.2 (proximal humerus)<br>S52.5 (distal radius)<br>M80.x0,1,2,3,4,6,7,9*) or (M 80.x* (including, among others, M80.99*, M80.08*)<br>AND NOT (Number 1 or Number 2 or S22.3 or S32.1 or S32.4 or S32.5 or S42.2 or S52.5 or M80.x0,1,2,3,4,6,7,9))
6	Forearm fracture	S52.5, S52.6. 52.8, 52.9, M80.x3*
7	Fracture of humerus	S42.2x, M80.x2*
8	Fracture of pelvis	S32.1, S32.4, S32.5
9	Fracture of neck of the femur	S72.0x except S72.08
10	Pertrochanteric fracture	S72.1
11	Large osteoporotic fracture	S22.01-06<br>S32.00<br>S32.01-05S 52.4<br>S72.0<br>S72.00

		S72.01-05<br>S72.1<br>S72.2<br>S52.4, S42.2<br>S42.2, S42.20, S42.21<br>S42.22 S42.23 S42.24<br>S42.29, M80.x2,3,5,8*
12	Osteoporotic fracture, any form	Number 1 or<br>Number 2 or<br>Number 3 or<br>M80.x* (including, among others, M80.99*, M80.08*)

An asterisk (*) indicates that at this position a modifier for any feature is allowed.

Table S2: Codes used from the Anatomical Therapeutic Chemical (ATC) Classification System

**Class**	**ATC code**
Calcium	A12A
Hormone therapy	G03
Vitamin D	A11C2 or A11C3
Bisphosphonates	M05BA
Denosumab	M05BX04
Tibolone	G03CX01
Raloxifene	G03XC01
Strontium ranelate	M05BX03
Parathormone	H05AA03
